# Dihydrocapsaicin Attenuates Blood Brain Barrier and Cerebral Damage in Focal Cerebral Ischemia/Reperfusion via Oxidative Stress and Inflammatory

**DOI:** 10.1038/s41598-017-11181-5

**Published:** 2017-09-05

**Authors:** Adchara Janyou, Piyawadee Wicha, Jinatta Jittiwat, Apichart Suksamrarn, Chainarong Tocharus, Jiraporn Tocharus

**Affiliations:** 1 Department of Anatomy, Faculty of Medicine, Chiang Mai University, Chiang Mai, 50200 Thailand; 2 Faculty of Medicine, Mahasarakham University, MahaSarakham, Thailand; 3 Department of Chemistry and Center of Excellence for Innovation in Chemistry, Faculty of Science, Ramkhamhaeng University, Bangkok, 10240 Thailand; 4 Department of Physiology, Faculty of Medicine, Chiang Mai University, Chiang Mai, 50200 Thailand

## Abstract

This study investigated the effect of dihydrocapsaicin (DHC) on cerebral and blood brain barrier (BBB) damage in cerebral ischemia and reperfusion (I/R) models. The models were induced by middle cerebral artery occlusion (MCAO) for 2 h followed by reperfusion. The rats were divided into five groups: sham, or control group; vehicle group; and 2.5 mg/kg, 5 mg/kg, and 10 mg/kg BW DHC-treated I/R groups. After 24 h of reperfusion, we found that DHC significantly reduced the area of infarction, morphology changes in the neuronal cells including apoptotic cell death, and also decreased the BBB damage via reducing Evan Blue leakage, water content, and ultrastructure changes, in addition to increasing the tight junction (TJ) protein expression. DHC also activated nuclear-related factor-2 (Nrf2) which involves antioxidant enzymes like superoxide dismutase (SOD) and glutathione peroxidase (GPx), and significantly decreased oxidative stress and inflammation via down-regulated reactive oxygen species (ROS), NADPH oxidase (NOX2, NOX4), nuclear factor kappa-beta (NF-ĸB), and nitric oxide (NO), including matrix metalloproteinases-9 (MMP-9) levels. DHC protected the cerebral and the BBB from I/R injury via attenuation of oxidative stress and inflammation. Therefore, this study offers to aid future development for protection against cerebral I/R injury in humans.

## Introduction

Stroke is the second leading cause of death worldwide. The number of deaths from stroke is projected to rise to 7.8 million in 2030^1–4^. Ischemic stroke is the most common cause of stroke, and it constitutes 80% of the cases that are caused from two main sources, thrombus and embolus^5, 6^. Occlusion of cerebral blood flow leading to depletion of energy and nutrients for cellular metabolism is what induces imbalance of osmotic gradients, membrane depolarization, and increasing of intracellular calcium, resulting in mitochondrial dysfunction and change in astrocytic plasticity. Nowadays, tissue plasminogen activator (tPA), a thrombolytic agent, is used to treat ischemic stroke in order to restore cerebral blood flow which would promote production of large amounts of reactive oxygen species (ROS), mainly from nicotinamide adenine dinucleotide phosphate (NADPH) oxidase or NOX, a family of transmembrane proteins^7–11^. In the stroke model, NOX2 and NOX4 are major sources of ROS which occurs 8–16 h after reperfusion^12^. NOX2 is expressed mainly in the membrane of endothelial cells, whereas NOX4 is expressed mainly in neurons^11^. Overproduction of ROS induces oxidative stress, which then activates the nuclear factor kappa-beta (NF-ĸB) signaling pathway to synthesize pro-inflammatory cytokines such as nitric oxide (NO), the tumor necrosis factor alpha (TNF-α), interleukin 6 (IL-6) which promotes neuronal inflammation, and matrix metalloproteinases-9 (MMP-9) which contribute to the breakdown of extracellular matrix and direct degrading of tight junction (TJ) proteins, leading, thus, to blood brain barrier (BBB) damage^13, 14^. During inflammation, cells maintain homeostasis by regulating the defense system. The major mechanism of cellular defense is nuclear-related factor-2 (Nrf2), an anti-inflammatory pathway which is associated with antioxidants that inhibit oxidative stress and anti-inflammation response. The activation of the Nrf2 signaling pathway triggers the translocation into the nucleus and then promotes transcription of its target genes to synthesize antioxidant enzymes such as superoxide dismutase (SOD) and glutathione (GSH)^13–15^. Thus, the activation of this system is important for protection against cerebral damage during I/R.

Capsaicin and dihydrocapsaicin (DHC, 8-methyl-N-vanillylnonanamide; N-[–4-hydroxy-3-methoxybenzyl]−8-methylnonanamide^6, 7^) are the two major active substances of capsaicinoids in chili peppers. Several lines of evidence have demonstrated that extracts from capsaicinoids have multiple pharmacological and physiological effects, including anti-cancer, anti-inflammation, antioxidant, anti-obesity, and pain relief^16–19^. Many previous studies have demonstrated that capsaicin has many pharmacological advantages, but few studies have previously mentioned the advantages as regards DHC^20–24^. DHC has been shown to pharmacologically induce hypothermia via TRPV1 channel agonism, thus providing neuroprotection in I/R mice^25^. Therefore, it is possible that DHC, a compound in capsaicinoids, may decrease oxidative stress and inflammation in I/R injury. In this study, we firstly demonstrate that DHC attenuates cerebral and BBB damage in I/R rat models via reducing oxidative stress and inflammation, and promoting the antioxidative pathway.

In this study, we investigate the protective effect of DHC on the BBB and cerebral damage from middle cerebral artery occlusion and reperfusion in Wistar rats by determining oxidative stress and inflammation. Our data suggest that DHC is a neuroprotective agent against cerebral ischemia and reperfusion via attenuation of BBB and cerebral damage, and warrants further evaluation.

## Results

### DHC improves neurological functional outcome and attenuates cerebral infarction

**Figure 1:**
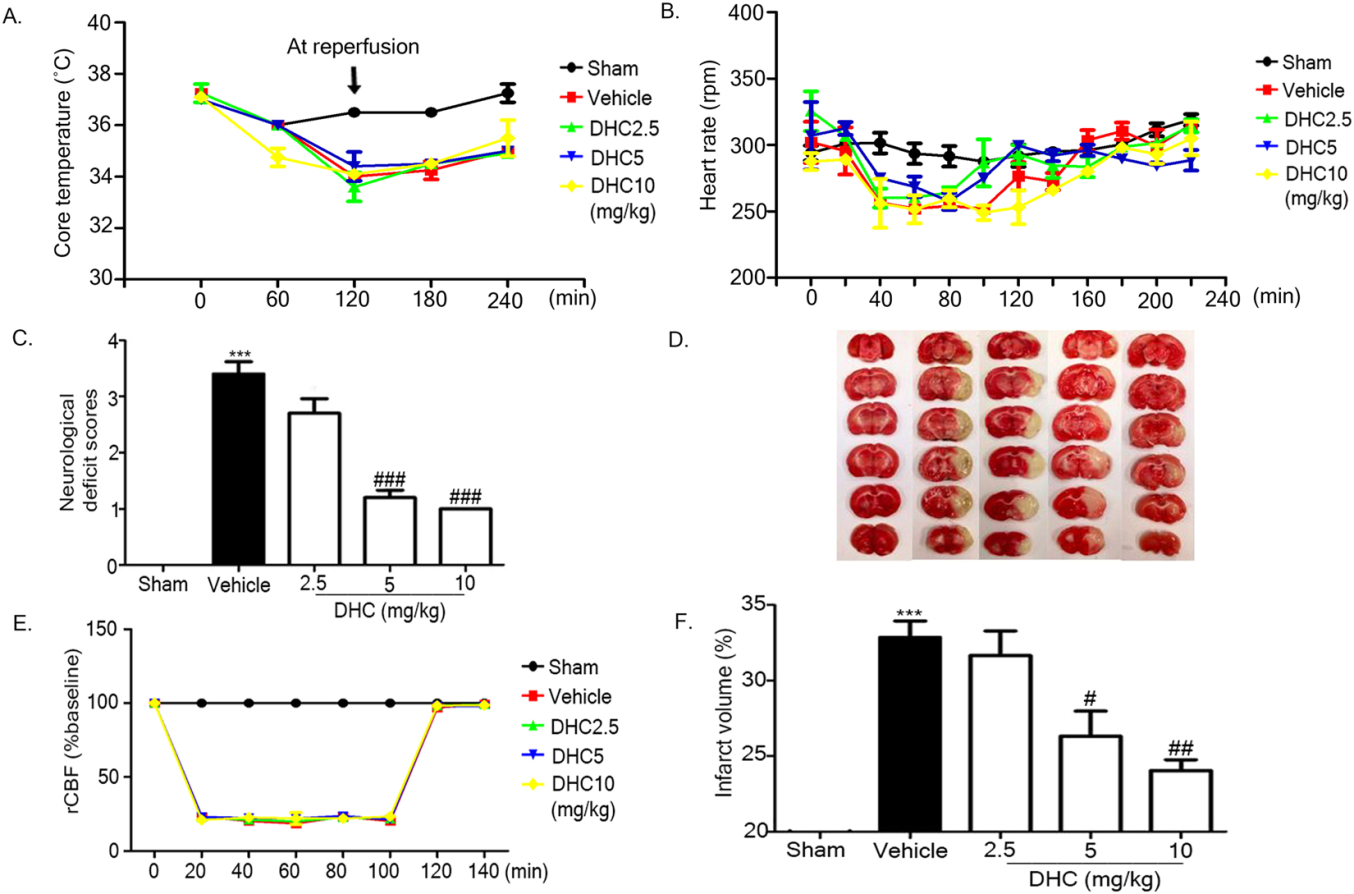
Decrease in neurological deficit scores and area of infarction in DHC-treated I/R. (**A** and **B**) Representative of core temperature and heart rate. **(C)** Representative of neurological deficit scores after 24 h reperfusion. (**D** and **F**) Percentage of infarct volume. (**E**) Regional cerebral blood flow was monitored before ischemia/reperfusion, during ischemia, and after reperfusion. Representative of TTC-stained coronal section after 24 h of reperfusion. The data are the mean ± S.E.M from three independent experiments (^***^ *p* < 0.001 compared with the sham group; and ^#^ *p* < 0.05, ^##^ *p* < 0.01, and ^###^ *p* < 0.001 compared with the vehicle group).

To investigate the protective effects of DHC in I/R models, DHC (2.5 mg/kg, 5 mg/kg, and 10 mg/kg) or the vehicle was intraperitoneally injected 15 min before reperfusion. DHC had no effects on core temperature and heart rate when compared with vehicle group (Figs 1A,1B). At 24 h after reperfusion, the neurological deficit scores were determined; it was found that the DHC treatment (5 mg/kg and 10 mg/kg) significantly decreased the neurological deficit scores compared with the vehicle (^###^ *p* < 0.001) (Fig. 1C). In addition, the region of cerebral blood flow (rCBF) was observed to have no significant changes between the I/R groups treated with the three doses of DHC and the vehicle during the ischemic period and after reperfusion (Fig. 1E). The infarct volume was determined by 2,3,5-triphenyltetrazolium chloride (TTC) staining: the area of infarction is represented as white in the ipsilateral hemisphere (Fig. 1D). The percentage of infarct volume was found to have increased in the vehicle group and significantly reduced in the DHC treatment groups (5 mg/kg and 10 mg/kg) compared with the vehicle group (^#^ *p* < 0.05) (Fig. 1F).

### DHC prevents morphology change and decreases apoptotic cell death

**Figure 2:**
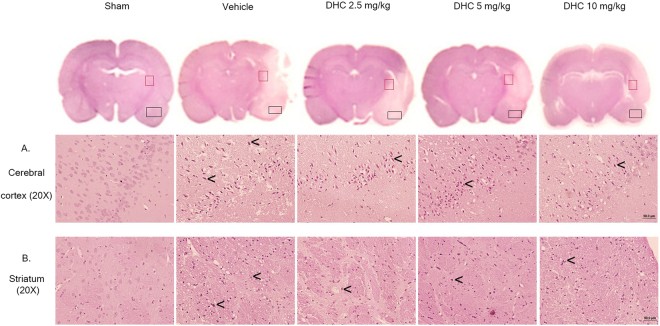
Effect of DHC on histopathology. (**A**) H&E-stained cerebral cortex of I/R brain after 24 h of reperfusion (20×). **(B**) Striatum (20×). The images were visualized with a light microscope (scale bar = 50 µm). The black arrow represents the pyknotic nucleus.

To further investigate the protective effects of DHC in I/R brain injury, the morphology changes were observed by hematoxylin and eosin (H&E) staining after 24 h of reperfusion. At the cerebral cortex, the neuronal cells became a pyknotic nucleus (black arrow) and vacuole around the nucleus, and this is widely represented in the vehicle group. The DHC treatment groups reduced the number of neuronal changes (Fig. 2A). Correspondingly, there were changes in morphology in the striatum (Fig. 2B). There were no morphological changes observed in the sham group.

**Figure 3:**
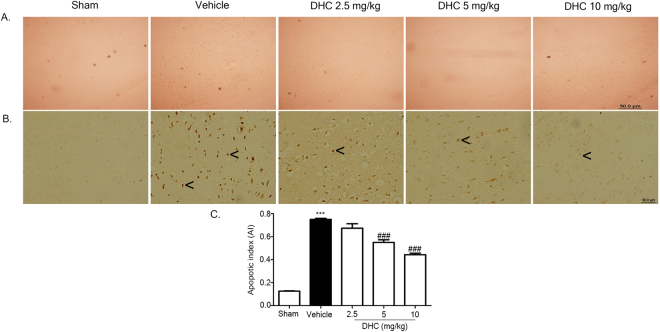
Effect of DHC on TUNEL staining in cerebral I/R rats. (**A** and **B**) Representative of TUNEL-stained apoptotic cell of I/R brain after 24 h of reperfusion, shown as positive brown particles (4× and 20×). (**C**) Apoptotic index (AI). The images were visualized with a light microscope (scale bar = 50 µm). The data are the mean ± S.E.M from three independent experiments (^***^ *p* < 0.001 compared with the sham group and ^###^ *p* < 0.001 compared with the vehicle group).

To investigate the neuroprotective effects of DHC against I/R via attenuation of the number of apoptotic cell deaths, TUNEL staining was performed to detect apoptotic cell death in the penumbra area at the cerebral cortex (Fig. 3A). The TUNEL positive cells were shown as dark brown particles as apoptotic cells. The sham group had no TUNEL positive cells. The apoptotic index in the DHC treatment groups (5 mg/kg and 10 mg/kg) had significantly reduced when compared with the vehicle group (^###^ *p* < 0.001) (Fig. 3B).

### DHC reduces BBB disruption via increasing expression of TJ proteins

**Figure 4:**
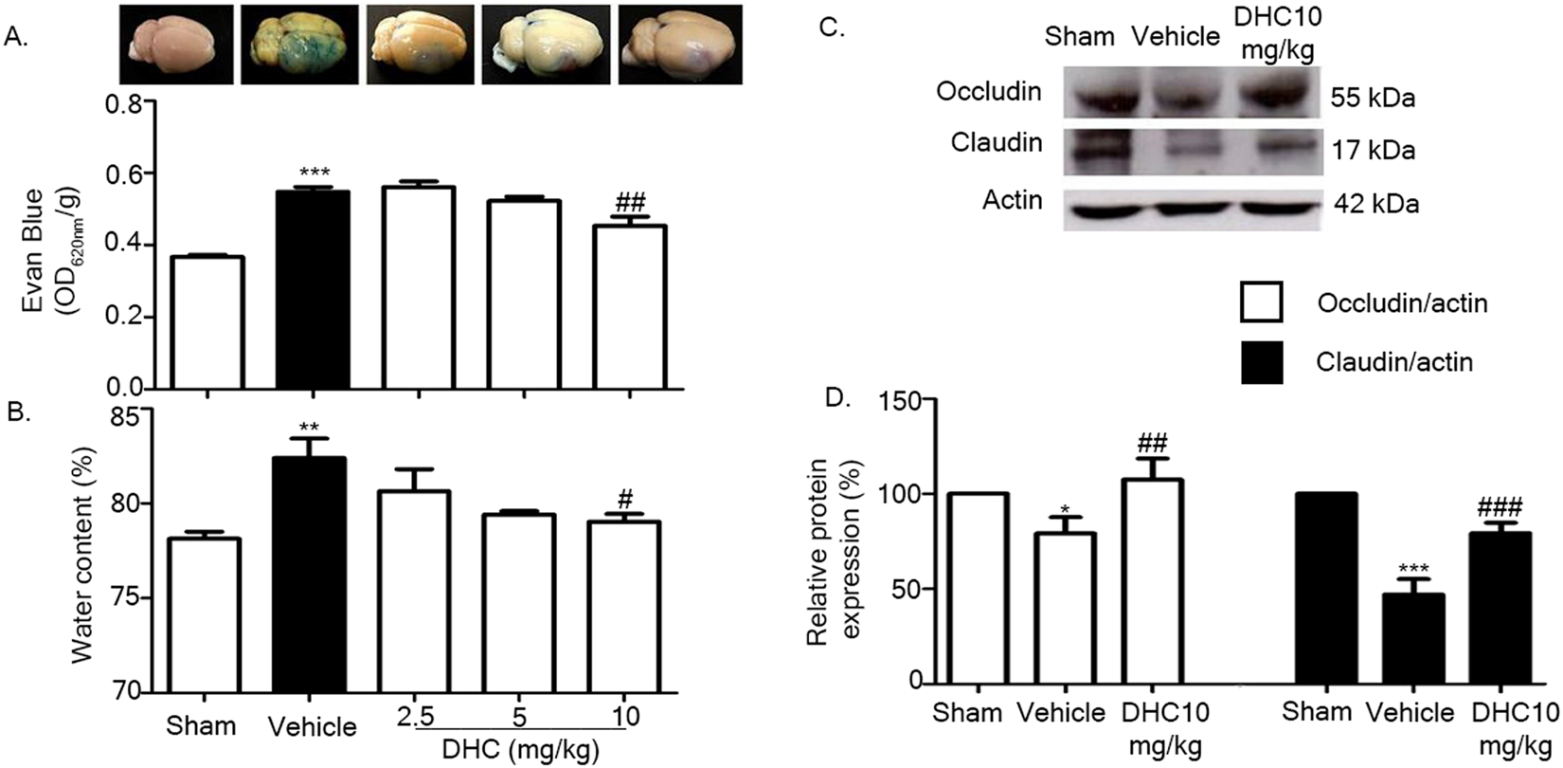
Administration of DHC protected BBB permeability and increased the level of TJ proteins. (**A**) The images represent the Evan Blue injected brain after 24 h of reperfusion, and the absorbance of the Evan Blue leakage is shown as OD_620nm_/g. (**B**) Percentage of brain water content. (**C**) Representative of western blot analysis of occludin and claudin in cerebral I/R rats at 24 h after reperfusion. (**D**) Quantitative analysis of protein expressions of occludin and claudin normalized using β-actin. The data are the mean ± S.E.M from three independent experiments (^*^ *p* < 0.05, ^**^ *p* < 0.01, and ^***^ *p* < 0.001 compared with the sham group; and ^#^ *p* < 0.05, ^##^ *p* < 0.01, and ^###^ *p* < 0.001 compared with the vehicle group).

The objective was to investigate the protective effects of DHC on the BBB in I/R models. The Evan Blue injection was performed. The BBB disruption was represented by the leakage of the Evan Blue color as dark blue in the ipsilateral brain tissue (Fig. 4A). The sham group was observed to have no leakage of the Evan Blue color. The severity of the BBB disruption was shown as OD_620nm_/g; the DHC treatment group (10 mg/kg) was observed to have significantly decreased the leakage of the Evan Blue color when compared with the vehicle group (^##^ *p* < 0.01) (Fig. 4A). The percentage of water content was determined to confirm the effects of DHC on the BBB in I/R models. The results showed an increasing in the water content in the vehicle group and a significant decrease in the same in the DHC group (10 mg/kg) (^#^ *p* < 0.05) (Fig. 4B), which is associated with the Evan Blue injection assay. Taking these results into consideration, DHC at the dose of 10 mg/kg was subsequently used to examine and conduct the study further.

To investigate the mechanism of DHC on BBB integrity in I/R models, western blotting was performed, as also to detect the expression of TJ proteins (occludin and claudin). The result showed that the expressions of both occludin and claudin in the vehicle group had significantly reduced compared with the sham group. The DHC treatment was observed to have significantly increased the expression of these proteins compared with the vehicle group (^#^ *p* < 0.05) (Fig. 4C).

### Effect of DHC on ultrastructure alterations in BBB in I/R models

**Figure 5:**
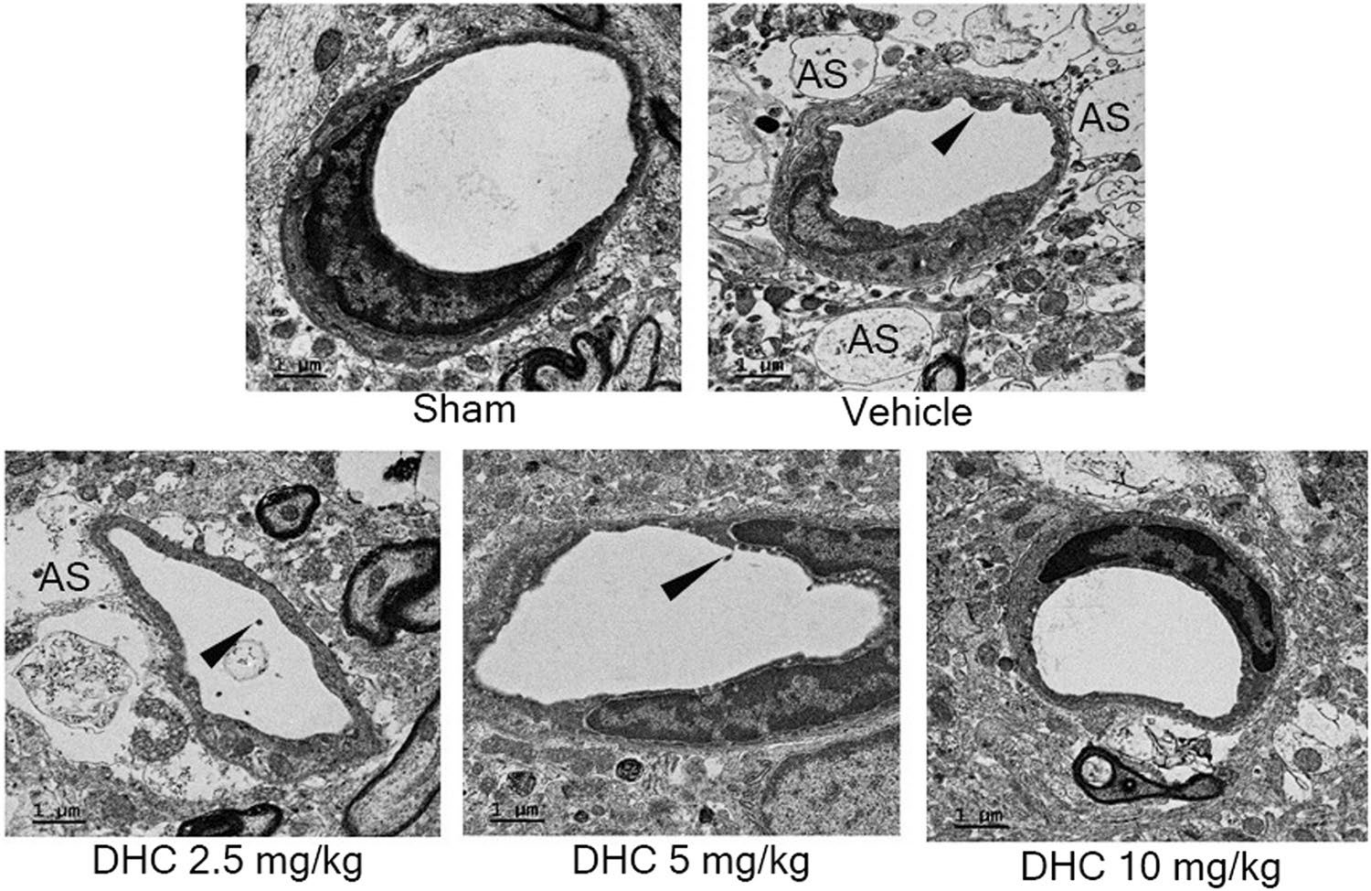
DHC-treated I/R reduced the ultrastructure changes in the BBB. Transmission electron micrographs showing the ultrastructure of the BBB in cerebral I/R rats at 24 h after reperfusion. Swelling of astrocytic foot plate (AS); black arrow shows microvillous formation. The images were visualized with TEM at a magnification of 6000× (scale bar = 1 µm).

To observe the protective effect of DHC on the ultrastructure of the BBB in I/R models, transmission electron microscopy (TEM) was performed, the brain samples were collected from penumbra area in cerebral cortex at the same level (3 samples per groups), fifteen capillaries (diameter 8–10 µm) were selected under JEM-2200FS TEM. The evaluation and taking a photograph were performed by technician who blinded to the treatment. The indicators of ultrastructure changes including alteration of lumen, numbers of microvillies, astrocytic foot plate swelling. Our results revealed that I/R caused ultrastructure changes in the BBB, astrocytic swelling (AS) around the endothelial cells, and alteration of the vascular lumen and the numbers of microvilli that were most present in the vehicle group, but that the changes were reduced in the DHC treatment group, especially in the DHC group with the dose 10 mg/kg which was found to be similar to the sham group (Fig. 5).

### DHC reduces NOX2 expression, NOX4 expression, and oxidative stress

**Figure 6:**
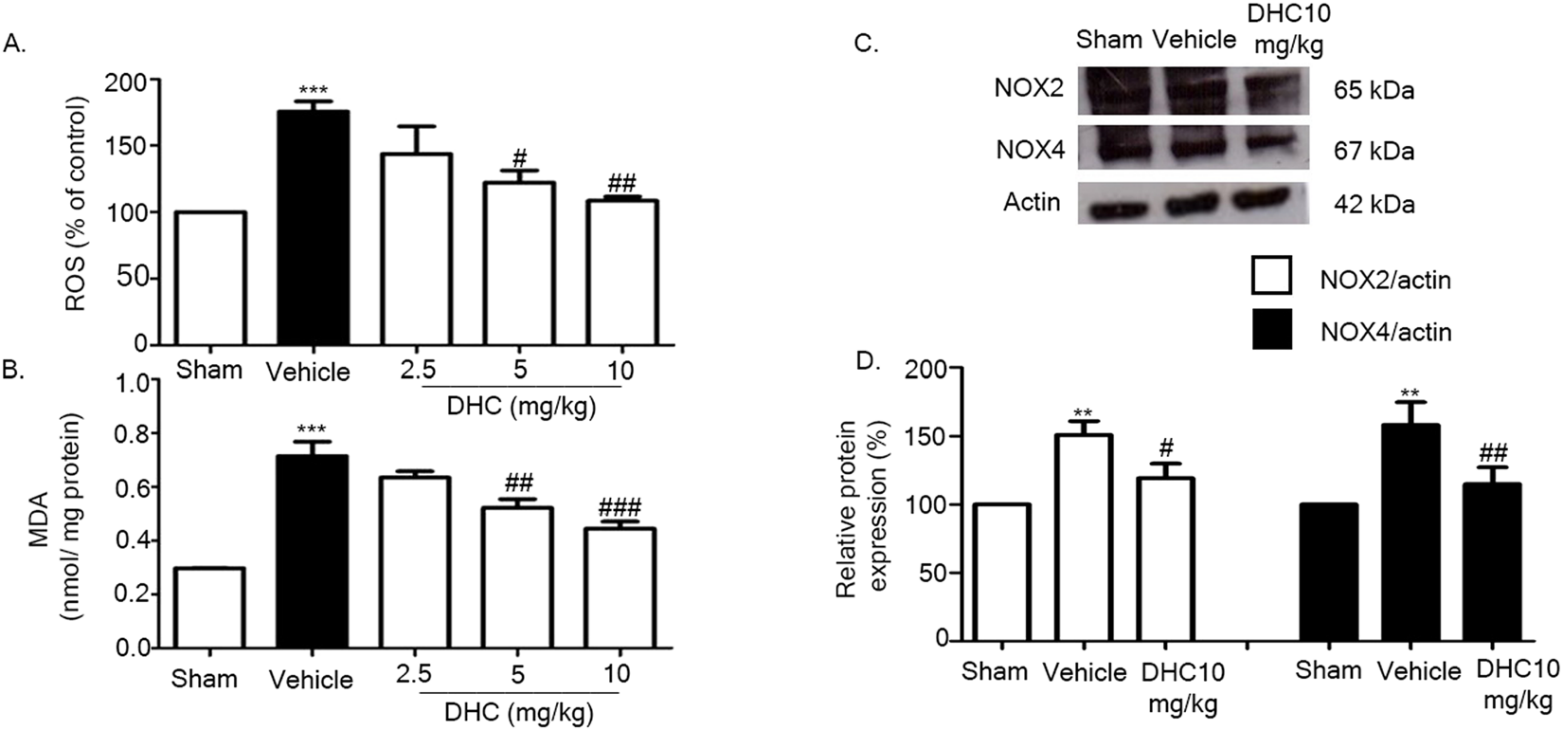
Administration of DHC attenuated oxidative stress via down-regulating the expressions of NOX2, NOX4, ROS, and lipid peroxidation. (**A**) Representative of ROS production. (**B**) Representative of MDA product of I/R brain after 24 h of reperfusion. (**C**) Representative of western blot analysis of NOX2 and NOX4 in cerebral I/R rats at 24 h after reperfusion. (**D**) Quantitative analysis of protein expression of NOX2 and NOX4 normalized using β-actin. The data are the mean ± S.E.M from three independent experiments (^**^ *p* < 0.01 and ^***^ *p* < 0.001 compared with the sham group; and ^#^ *p* < 0.05, ^##^ *p* < 0.01, and ^###^ *p* < 0.001 compared with the vehicle group).

I/R increases the overproduction of ROS in the central nervous system which is mainly produced from NOX2 and NOX4^10, 12^. To detect the ROS production, we used the DCF assay. Our results found that ROS was widely expressed in the vehicle group, but significantly reduced in the DHC treatment groups (5 mg/kg and 10 mg/kg) (^#^ *p* < 0.05, ^##^ *p* < 0.01) (Fig. 6A). Then, the ROS production induced lipid peroxidation and oxidative stress^26, 27^. We performed the malondialdehyde, or MDA, assay to detect lipid peroxidation end products. Our results suggest that I/R induced the MDA products. In addition, administration of DHC (5 mg/kg and 10 mg/kg) also significantly reduced the MDA products in comparison with the vehicle group (^##^ *p* < 0.01 and ^###^ *p* < 0.001) (Fig. 6B).

To investigate whether NADPH promotes ROS production and oxidative stress, we determined the protein expressions of NOX2 and NOX4 after I/R injury. We found that the expressions of NOX2 and NOX4 had increased in the vehicle group, and that the administration of DHC had significantly reduced the expressions of NOX2 and NOX4 (^#^ *p* < 0.05) (Fig. 6C–D).

### DHC suppresses NF-κB signaling pathway

**Figure 7:**
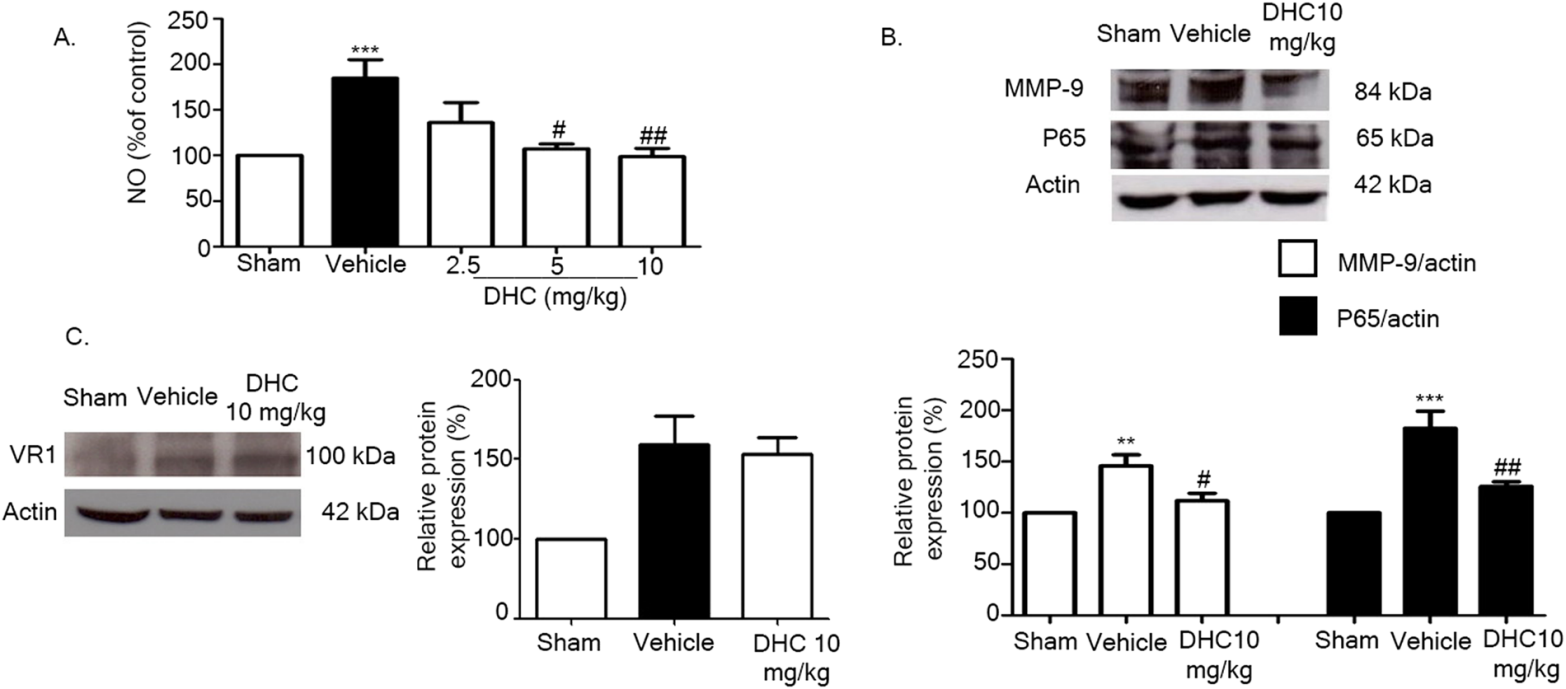
Effect of DHC on the inflammatory pathway through down-regulating NO production, NF-κB (p65 subunits), and MMP-9 level. (**A**) NO production is shown as % of control after 24 h of reperfusion. (**B**) Representative of western blot analysis of MMP-9 and p65 in cerebral I/R rats at 24 h after reperfusion. Quantitative analysis of protein expressions of MMP-9 and p65 normalized using β-actin. (**C**) Representative of western blot analysis of TRPV1 in cerebral I/R rats at 24 h after reperfusion. Quantitative analysis of protein expressions of TRPV1 normalized using β-actin. The data are the mean ± S.E.M from three independent experiments (^**^ *p* < 0.01 and ^***^ *p* < 0.001 compared with the sham group; and ^#^ *p* < 0.05 and ^##^ *p* < 0.01 compared with the vehicle group).

To investigate the mechanism of DHC on I/R injury under inflammatory signaling pathways, the Griess reaction assay was performed to detect NO production; we found that the level of nitrite production had increased in the vehicle group and significantly decreased in the DHC treatment groups (^#^ *p* < 0.05 and ^#^ *p* < 0.01) (Fig. 7A). Then, western blot analysis was performed to detect the expression of p65 (NF-κB subunits) and MMP-9. The results demonstrated that the levels of p65 and MMP-9 had increased in the vehicle group and significantly decreased in the DHC treatment group (^#^ *p* < 0.05) (Fig. 7B). To investigate whether effects of DHC on TRPV1 expression, western blot analysis was performed. The results showed that DHC had no effects with TRPV1 expression (Fig. 7C).

### DHC promotes Nrf2 signaling pathway

**Figure 8:**
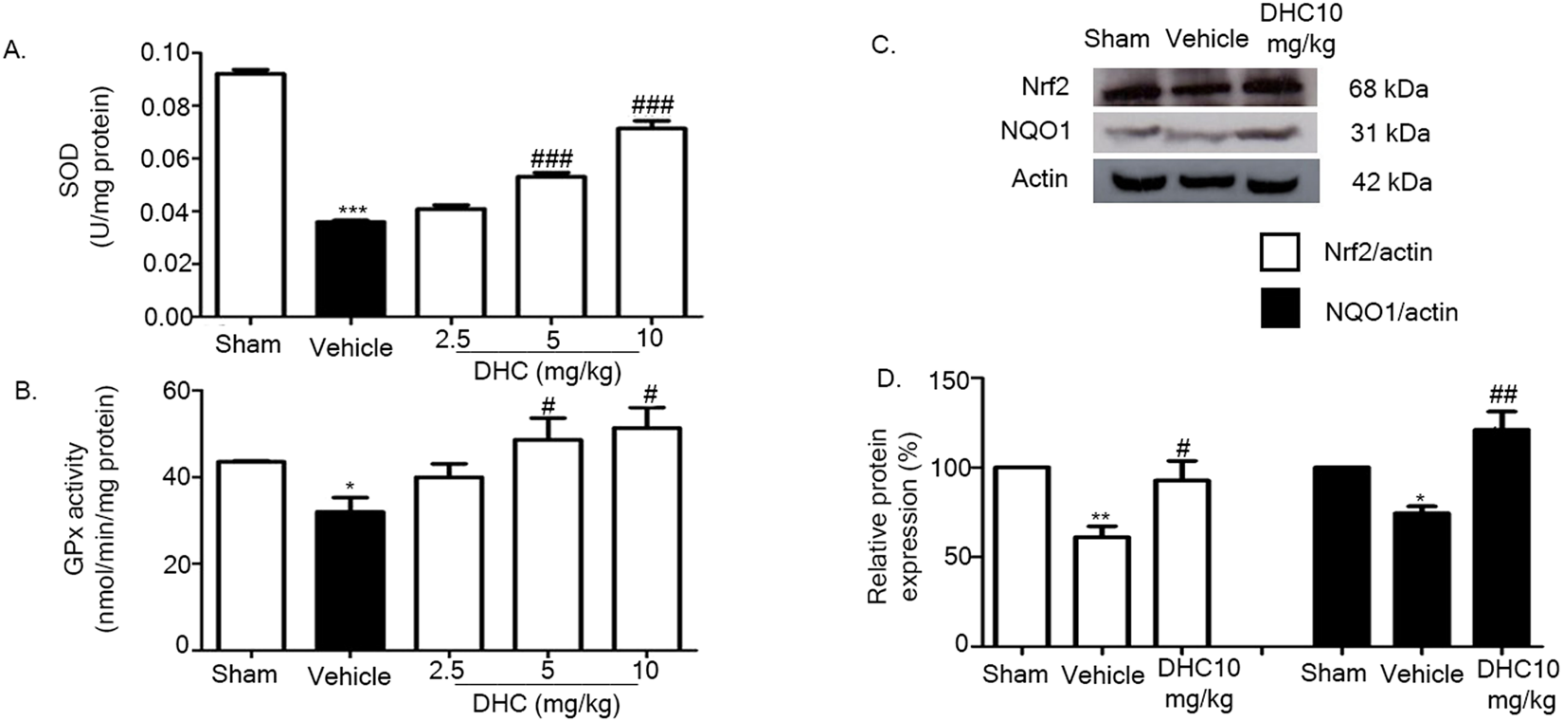
Effect of DHC on the Nrf2 signaling pathway via upregulating Nrf2 and NQO1 levels and promoting SOD and GPx activities. (**A** and **B**) SOD and GPx activities in cerebral I/R rats after 24 h of reperfusion. (**C**) Representative of western blot analysis of Nrf2 and NQO1 in cerebral I/R rats at 24 h after reperfusion. (**D**) Quantitative analysis of protein expressions of Nrf2 and NQO1 normalized using β-actin. The data are the mean ± S.E.M from three independent experiments (^*^ *p* < 0.05, ^**^ *p* < 0.01, and ^***^ *p* < 0.001 compared with the sham group; and ^#^ *p* < 0.05, ^##^ *p* < 0.01, and ^###^ *p* < 0.001 compared with the vehicle group).

Nrf2 is a major mechanism of cellular defense, an anti-inflammatory pathway associated with antioxidant enzymes such as GSH and SOD^13^. To investigate SOD activity, the SOD activity assay was performed. Our results found that I/R decreased the SOD activity, but the DHC treatment groups (5 mg/kg and 10 mg/kg) were found to have significantly increased SOD activity when compared with the vehicle group (^###^ *p* < 0.001) (Fig. 8A). Then, we investigated the GPx activity; our data showed that GPx activity had reduced in the vehicle group and significantly increased in the DHC treatment groups (5 mg/kg and 10 mg/kg) (^#^ *p* < 0.05) (Fig. 8B).

To explore the mechanism by which DHC brings out its anti-inflammation effects, the protein levels of Nrf2 and NQO1 in the I/R cortex were determined by western blotting. As shown in Fig. 8C–D, the expression of Nrf2 decreased in the vehicle group but significantly increased in the DHC group (^#^ *p* < 0.05), as did the expression of NQO1.

## Discussion

Capsaicin and DHC are the two major components of capsaicinoids in chili peppers. Previous works have reported that capsaicin acts as an anti-inflammation mediated in both transient receptor potential vanilloid 1 (TRPV1) and the receptor-independent pathway^20^. DHC has been shown to pharmacologically induce hypothermia via TRPV1 channel agonism, thereby providing neuroprotection in I/R mice^25^. However, the ability of DHC to attenuate BBB damage following I/R injury has not yet been studied. Hence, in this study, we firstly investigate the underlying mechanism of DHC to attenuate cerebral and BBB damage in I/R rat models mediated by NF-κB and Nrf2 signaling pathways.

Ischemia induces complex metabolic occurrences that lead to irreversible damage and neuronal cell death in the ischemic core. The collateral cerebral artery near the cerebral artery occlusion can help to reduce ischemia in the penumbra area^7^. Our results demonstrate that administration of DHC attenuates neurological deficit scores and areas of cerebral infarction. Restoration of cerebral blood flow (reperfusion) results in increased concentration of oxygen in the blood and production of ROS. Overproduction of ROS is mainly from enzymatic sources like xanthine oxidase, NOX, mitochondria, and NO synthase^28^. Previous studies have reported that NOX2 and NOX4 are the main sources of ROS in cerebral I/R injury^12^. Then, ROS induces lipid peroxidation and protein degradation, resulting in oxidative stress. Therefore, the inhibition of NOX can attenuate ROS production and lipid peroxidation, and also decrease the area of infarction^12^. Our results show that DHC treatment significantly reduces the expression levels of NOX2 and NOX4, in addition to reducing ROS production and lipid peroxidation. Consequently, oxidative stress can activate the NF-κB signaling pathway, leading to the release of pro-inflammatory cytokines, which would induce BBB disruption and vasogenic edema. The critical pro-inflammatory enzyme that plays an important role in BBB disruption is iNOS which generates NO^13^ and MMP-9. Furthermore, the activity of MMP-9 is breaking down of the BBB via directly degrading TJ proteins like claudin and occludin, which initiates vasogenic edema^14^. Xu *et al*., 2015, demonstrated that suppressing the expression of MMP-9 mediates toward decreasing the degradation of claudin-5 and occludin, and also reducing the BBB permeability in cerebral I/R injury^29^. Capsaicin and DHC have been shown to have anti-inflammatory properties via suppression of NF-κB expression and pro-inflammatory cytokines (IL-1β, IL-6, and TNFα)^16, 24, 30^. Our results demonstrate that DHC also suppresses the NF-κB signaling pathway by decreasing the expression of p65 (NF-κB subunits), MMP-9, and NO production. In addition, DHC treatment of I/R was found to increase the levels of TJ proteins like occludin and claudin. This finding correlates with the study of the Evan Blue injection assay that the vehicle group showed mostly leakage of the Evan Blue color which indicated BBB disruption^29, 31^. However, this effect can be reduced in DHC-treated groups in the same way as the ultrastructure changes from TEM, cerebral microvascular alteration such as vascular lumen alteration, and forming of microvilli; a proportion of astrocytic swelling^32^ is also revealed in cerebral I/R injury and it is seen as reduced in DHC-treated groups. These results indicate that DHC is able to protect against BBB disruption in cerebral I/R injury and also reduce brain edema. Moreover, oxidative stress leads to an unsterilized state of the Nrf2/Keap1 complex and promotes antioxidant enzymes including SOD, CAT, and GSH^13, 15^. SOD enzymes can catalyze superoxide ions into H_2_O and O_2_; GPx that coupled reaction with glutathione reductase to provide reduced glutathione into oxidized glutathione which is against oxidative stress^13, 33^. A previous study suggested that activation of Nrf2 leads to the promotion of antioxidative enzymes which decrease neurological deficit scores, infarct volume, and oxidative stress^34^ related to our results; the DHC treatment was observed to promote the protein expressions of Nrf2 and NQO1, which involves SOD production, and also significantly increase the SOD activity and the GPx activity when compared with the vehicle group. These results suggest that DHC enhances the antioxidative mechanism against oxidative stress in cerebral I/R. Our results also showed the histological changes in the cerebral cortex and the striatum in the I/R brain, with the neuronal cells presenting a pyknotic nucleus (dense body and irregular nucleus), cell shrinkage (vacuoles around the cells), and decrease in the number of neuronal cells which correlated with the apoptotic cell death in the TUNEL assay^35^. DHC administration is able to improve histology changes in the cerebral and the striatum in the penumbra area and also decrease the number of apoptotic cell deaths.

In conclusion, our findings suggest that DHC-treated cerebral I/R rats attenuate cerebral and BBB damage through inhibition of oxidative stress and inflammatory pathways. The protective effects of DHC are related to its antioxidant and anti-inflammatory actions. Our study will help to discover novel therapeutic techniques based on DHC in the treatment of ischemic stroke.

## Methods

### Antibodies and reagents

DHC was purchased from Anyang General International (Henan, China) and the purity was 96.7% (from HPLC analysis). Anti-NOX2, anti-NOX4, anti-Nrf2, anti-NQO1 and anti-VR1 were purchased from Abcam (Abcam, MA, USA). Anti-MMP-9 were purchased from cell signaling (Danvers, MA, USA). Anti-NF-kB, anti-claudin, anti-occludin, and anti-β-actin were purchased from Millipore (Millipore, MA, USA). Anti-mouse IgG peroxidase conjugated secondary antibody and anti-rabbit IgG peroxidase conjugated secondary antibody were purchased from Merck Millipore (MA, USA). Commercial kits used for determining SOD and GPx activities were obtained from Cayman (Cayman Chemicals, Ann Arbor, MI, USA). All other reagents were obtained from Sigma (St. Louis, MO).

### Animals and drug administration

Male Wistar rats weighting 280–300 g were purchased from the National Laboratory Animal Center, Mahidol University, Salaya, Nakornpathom, Thailand. The animals were housed under restriction of controlled temperature (25 ± 1 °C) and light–dark cycles (12 h and 12 h). They had free access to food and water. All experimental procedures were approved by the Institutional Animal Care and Use Committee at the Faculty of Medicine, Chiang Mai University (Permitted number: 33/2559), in compliance with NIH guideline. Briefly, the animals were randomized and divided into five groups: (1) sham, or control, group; (2) vehicle group; (3) DHC 2.5 mg/kg BW; (4) DHC 5 mg/kg BW; and (5) DHC 10 mg/kg BW. DHC was dissolved in dimethyl sulfoxide (DMSO) and diluted in 2-hydroxyethyl cellulose, in the vehicle group also injected the same volume of 2-Hydroxyethyl cellulose (final concentration of DMSO was 1%) by intraperitoneal administration 15 min before cerebral reperfusion (except the sham group).

### Induction of middle cerebral artery occlusion and reperfusion model

Focal cerebral ischemia was performed through middle cerebral artery occlusion (MCAO) by using a modified intraluminal technique^36^. Briefly, the rats were anesthetized by intraperitoneal injection with zoletil (30 mg/kg) and xylazine (10 mg/kg). After the animals had achieved complete unconsciousness, they were turned to adopt the supine position on a heating pad for control, with the body temperature at 37.0 ± 0.5 °C, and then fixed with an adhesive tape. The animals were incised in the midline of the neck and the soft tissues were retracted. The right common carotid artery (CCA) was identified, and it was followed toward the rostral portion which bifurcated into the external carotid artery (ECA) and the internal carotid artery (ICA). The intraluminal filament (Doccol Corp., Sharon, MA, USA) was inserted past the ECA stump into the ICA (17–19 mm) until a slight resistance was felt. At this moment, the filament was blocked by the origin of the right MCA. The core temperature and heart rate were detected before, during and after operation. After 2 h, the filament was withdrawn carefully to allow MCA reperfusion. After surgery, anesthesia was discontinued and animals were maintained by heating pad for 2 h after reperfusion and also evaluated core temperature every hour for 4 h then, the animal were transferred to a room temperature environment (25 ± 1 °C) until sacrificed.

### Cerebral blood flow by laser Doppler flowmetry

Laser Doppler flowmetry (AD Instruments, Dunedin, New Zealand) was used to observe the regional cerebral blood flow (rCBF) in the territory of the middle cerebral artery (5 mm lateral and 1 mm posterior from the bregma) during pre-ischemia, ischemia, and reperfusion to confirm the completeness of the cerebral ischemia/reperfusion procedure.

### Measurement of neurological deficit scores

Behavioral tests were performed after 24 h of reperfusion. A five-point grading scale of neurological deficits was used, as described by Longa and coworkers in 1989^36^. The five-point scale was as follows: 0 = no neurological deficits, 1 = failure to extend contralateral forepaw fully, 2 = circling to the ipsilateral side when held by the tail, 3 = falling to contralateral side, and 4 = did not walk spontaneously and has depressed level of consciousness.

### Measurement of infarct volume

After the assessment of neurological elevation, the animals were sacrificed under deep anesthesia. The rat brains were removed and dissected into six slices (of 2 mm thickness) in the coronal plane beginning from the posterior of occipital lobe. The brain slices were stained with 2% solution of 2,3,5-triphenyltetrazolium chloride (TTC) at 37 °C for 20 min, and then fixed in buffered formalin. Images of the stained sections were taken and quantified by the Image J^®^ software. The relative infarct volume was obtained with correction for edema as 100% × [contralateral hemisphere volume − non-infarct ipsilateral hemisphere volume]/contralateral hemisphere volume.

### Detection of apoptotic cell death (TUNEL assay)

TUNEL staining was used to detect apoptotic cell death on the basis of DNA fragmentation. After 24 h of reperfusion, the cerebral cortex was fixed in 4% formaldehyde, regularly embedded in paraffin, and then sectioned at a thickness of 4 μm. The sections were deparaffinized and rehydrated. After that, TUNEL staining was performed according to the manufacturer’s instructions on the TUNEL assay kit (Roche Diagnotics Corp., Indianapolis, IN), with the nuclei stained in brown particles. The tissues were observed under light microscopy (Olympus AX70, Japan). The coronal section of cerebral cortex at the same level (five fields, ×20) were randomly selected, the number of apoptotic cells (positive brown particles) and the number of total cells of the five adjacent sections were counted. The average value was used to calculated the apoptotic index (Apoptosis index (AI) = number of positive cells/number of total cells).

### Detection analysis for BBB integrity

The BBB integrity was evaluated by using Evans Blue injection. In brief, after 24 h of reperfusion, the animals were immediately anesthetized and injected with 2% Evans Blue solution (4 ml/kg) by intravenous injection into the jugular vein. After 30 min of circulation of the Evan Blue solution, the animals were perfused intracardially under deep anesthesia with cold phosphate-buffered saline (PBS, pH 7.4). The brains were removed, separated, and homogenized in dimethyl sulfoxide, and then incubated at 50 °C for 2 h. The samples were centrifuged at 12,000 g for 30 min, the supernatants were collected, and the absorbance was measured at 620 nm by using a microplate reader (BioTek Instruments Inc, Winooski, VT, USA).

### Histology analysis

At 24 h after reperfusion, the animals were anesthetized and perfused intracardially with an isotonic sodium chloride solution, followed by 4% (w/v) paraformaldehyde in 0.1 M sodium phosphate buffer (pH 7.4). The brains were immediately removed and fixed for 48 h in 4% (w/v) paraformaldehyde. After that, the brains were embedded in paraffin and coronal sections (of 4 μm thickness) were sliced and stained with H&E. The cerebral cortex and the striatum at the same level of each groups were observed the morphology changes under a light microscope (Olympus AX70, Japan).

### Transmission electron microscopy (TEM)

After 24 h of reperfusion, the animals were perfused with PBS solution and the brains were fixed with 4% paraformaldehyde (PFA). For TEM analysis, the brain tissues were minced into 1 mm × 1 mm × 1 mm sized pieces and fixed with 2.5% glutaraldehyde in 0.1 phosphate buffer (pH 7.3) at 4 °C overnight. After dehydration, the samples were impregnated with epoxy resin and sectioned. The sections were double stained with lead citrate and uranyl acetate. The images were obtained using transmission electron microscopy (JEM-2200FS TEM).

### Measurement of ROS production (DCF assay)

The intracellular ROS was measured by using the oxidation-sensitive 2′,7′-dichlorodihydrofluorescein diacetate (DCFH-DA) dye. After 24 h of perfusion, the brains were removed and cut into coronal sections at the same level (2 µm). Preparation for total lysis buffer with protease inhibitor cocktail, 300 µl of lysis buffer/100 mg of sample, was added. The samples were homogenized and centrifuged at 12,000 g for 10 min at 4 °C and the supernatant was collected. A volume of 50 µl of the supernatant was placed in a 96-well plate and incubated with 10 µl of DCFH-DA solution for 25 min in the dark. The samples were measured by using a fluorescent microplate reader (DTX800, Beckman Coulter, Austria) at an excitation wavelength of 485 nm and an emission wavelength of 532 nm.

### Measurement of lipid peroxidation (MDA assay)

MDA was measured by the calorimetric assay. Briefly, the brains were removed and collected with 250 µl of cold assay buffer, and then homogenized. The sample was mixed with 10 µl of butylated hydroxytoluene in methanol and 250 µl of 1 M phosphoric acid, 250 µl of 2-thiobarbituric acid was added and mixed to this, and the mixture was incubated at 60 °C for 1 h. After the incubation, the samples were centrifuged at 10,000 g for 5 min. The supernatant was transferred into a 96-well plate and the absorbance was measured at 532 nm using a microplate reader (BioTek Instruments Inc, Winooski, VT, USA).

### Measurement of SOD activity

The brain tissues were collected and homogenized in the lysis buffer. The lysates were centrifuged at 12,000 rpm for 10 min at 4 °C, and the supernatants were collected. The SOD activity was performed by following the instructions on a superoxide dismutase assay kit (Cayman Chemical Company, Ann Arbor, MI). The samples and the SOD standard were placed in a 96-well plate, with 20 µl/well, and 160 µl of the working reagent was added per well. A volume of 20 µl/well of xanthine oxidase (XO) enzyme was added, and it was incubated for 30 min at 37 °C in the dark. The supernatant was measured by using a microplate reader (BioTek Instruments Inc, Winooski, VT, USA) at an absorbance of 450 nm.

### Measurement of glutathione peroxidase (GPx) activity

After 24 h of reperfusion, the brain tissues were collected and homogenized in the lysis buffer. The lysates were centrifuged at 12,000 rpm for 10 min at 4 °C, and the supernatants were collected. The GPx activity was measured using a commercially available kit (Cayman Chemical Company, Ann Arbor, MI) by following the protocol. The GPx activity was measured by using a microplate reader (BioTek Instruments Inc, Winooski, VT, USA) at 340 nm every minute for five points.

### Detection of NO production (Griess reaction assay)

The NO production was measured by the Griess reaction assay. After 24 h of reperfusion, the brain tissues were collected and homogenized in the lysis buffer. The lysates were centrifuged at 12,000 rpm for 10 min at 4 °C. A volume of 10 µl of the supernatant of each of the groups was placed in a 96-well plate and mixed with an equal volume of the Griess reagent (a mixture of 1% sulfanilamide in 5% phosphoric acid and 0.1% N-1-naphthylethylenediamine dihydrochloride) for 10 min. The absorbance was measured at 540 nm by using a microplate reader (BioTek Instruments Inc, Winooski, VT, USA).

### Western blot analysis

The brain tissues were homogenized and the total protein concentrations were measured using the Bradford protein assay (Bio-Rad Laboratories, Hercules, CA, USA). Equal amounts of proteins (25 μg) were loaded into the electrophoresis chamber in 10–15% SDS-PAGE and transferred to a PVDF membrane (Immobilon-P, Millipore, Bedford, MA, USA). The blots were blocked for 3 h at room temperature in a fresh blocking buffer (containing 5% skim milk in 0.1% Tween-20 in Tris-buffered saline, pH 7.4). The membranes were incubated with the primary antibodies (anti-NOX2 [1:1,000], anti-NOX4 [1:1,000], anti-Nrf2 [1:1,000], anti-NQO1 [1:1,000], anti-MMP-9 [1:1,000], anti-NF-kB [1:1,000], anti-VR1 [1:1,000], anti-claudin [1:2,000], and anti-occludin [1:2,000]) at 4 °C overnight. After washing with the Tris-buffer saline and Tween-20 (TBST), the membranes were incubated with anti-rabbit IgG or anti-mouse peroxidase-conjugated secondary antibody. Finally, the membrane was incubated with Immobilon Western (Millipore, MA, USA) and exposed to an X-ray film. The densitometric quantification was analyzed by using the Image-J® software. The reference gene β-actin was used for normalization.

### Statistic analysis

All values are presented as mean ± S.E.M. Statistical analysis was carried out using one-way analysis of variance (ANOVA) for comparisons between groups, followed by Dunnett’s post hoc test. A *P* value < 0.05 was considered as a statistically significant difference between the experimental and the control groups. All the experiments were carried out three times.

## Electronic supplementary material

Supplementary data
