# Supplementary material for: Dihydrocapsaicin Attenuates Blood Brain Barrier and Cerebral Damage in Focal Cerebral Ischemia/Reperfusion via Oxidative Stress and Inflammatory
Source: Sci Rep. 2017 Sep 5;7:10556. doi: 10.1038/s41598-017-11181-5 (PMC5585260; doi:10.1038/s41598-017-11181-5)
Supplement: Supplementary file 1 — Supplementary data [file 41598_2017_11181_MOESM1_ESM.doc]

**Dihydrocapsaicin Attenuates Blood Brain Barrier and Cerebral Damage in Focal Cerebral Ischemia/Reperfusion via Oxidative Stress and Inflammatory**

*Adchara Janyou^1^, Piyawadee Wicha^1^, Jinatta Jittiwat^2^, Apichart Suksamrarn^3^, Chainarong Tocharus^1^, and Jiraporn Tocharus^4,*^*

*^a^Department of Anatomy, Faculty of Medicine, Chiang Mai University, Chiang Mai 50200, Thailand*

*^b^Department of Chemistry and Center of Excellence for Innovation in Chemistry, Faculty of Science, Ramkhamhaeng University, Bangkok 10240, Thailand*

*^c^Department of Physiology, Faculty of Medicine, Chiang Mai University, Chiang Mai 50200, Thailand*

^*^Corresponding author at: Department of Physiology, Faculty of Medicine, Chiang Mai University, Chiang Mai 50200, Thailand.

Tel: (6653) 945362; Fax: (6653) 945365

*E-mail address*: jtocharus@gmail.com (J. Tocharus).

**Supplemental Figure Legends**

**Figure S1.** Representative of multiple exposure of the blots in figure 4 include occludin, claudin and actin in I/R rat after 24 h reperfusion.

**Figure S2.** Representative of multiple exposure of the blots in figure 6 include NOX2, NOX4 and actin in I/R rat after 24 h reperfusion.

**Figure S3.** Representative of multiple exposure of the blots in figure 6 include MMP9, P65 and actin in I/R rat after 24 h reperfusion.

**Figure S4**. Representative of multiple exposure of the blots in figure 6 include Nrf2, NQO1 and actin in I/R rat after 24 h reperfusion.

**Figure S5**. Representative of multiple exposure of the blots in figure 7 include TRPV1 and actin in I/R rat after 24 h reperfusion.


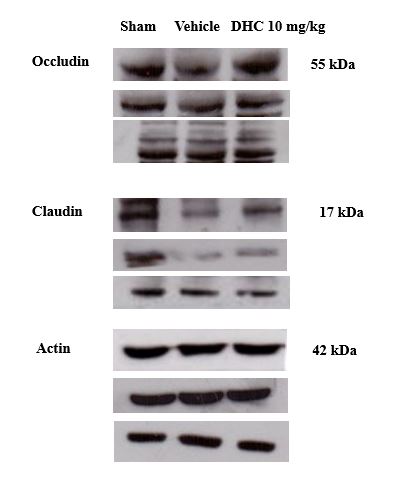


**Figure S1.**


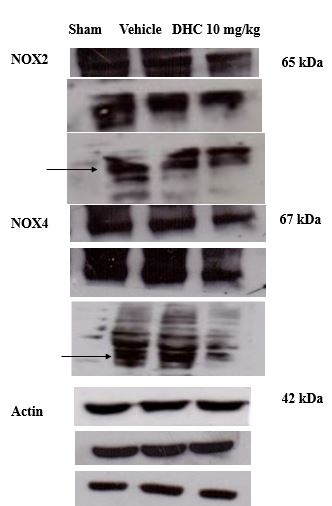


**Figure S2.**


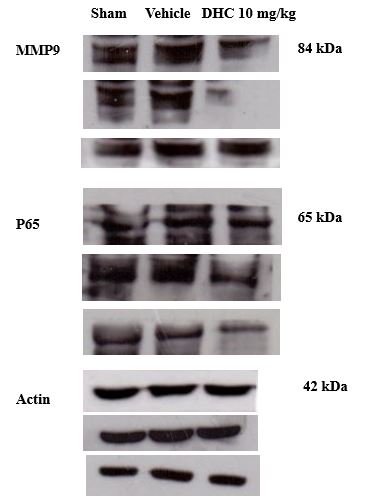


**Figure S3.**


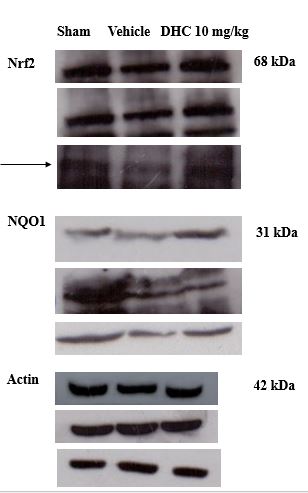


**Figure S4.**


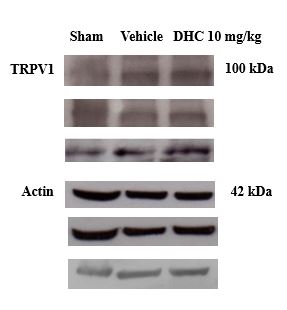


Figure S5.
